# Screening and management of hospital hyperglycemia in non-critical patients: a position statement from the Brazilian Diabetes Society (SBD)

**DOI:** 10.1186/s13098-025-01585-z

**Published:** 2025-02-12

**Authors:** Emerson Cestari Marino, Denise Momesso, Marcos Tadashi Kakitani Toyoshima, Maria Fernanda Ozorio de Almeida, Beatriz D. Schaan, Leandra Anália Freitas Negretto, Augusto Cezar Santomauro Junior, Priscilla Cukier, Paulo Roberto Rizzo Genestreti, Alina Coutinho Rodrigues Feitosa, Jorge Eduardo da Silva Soares Pinto, Rogerio Silicani Ribeiro, Rodrigo Nunes Lamounier, Ruy Lyra, Marcello Casaccia Bertoluci

**Affiliations:** 1 Curitiba Diabetes Center, Curitiba, Brazil; 2 Endocrinology Service, Universidade Federal Do Estado Do Rio de Janeiro, Rio de Janeiro, Brazil; 3 Endocrine Oncology Unit, Instituto Do Câncer Do Estado de São Paulo Octavio Frias de Oliveira, Hospital das Clínicas da Faculdade de Medicina da Universidade de São Paulo, São Paulo, Brazil; 4 Hospital das Clínicas da Faculdade de Medicina da Universidade de São Paulo, São Paulo, Brazil; 5 Faculdade de Medicina da Universidade Federal Do Rio Grande Do Sul, Porto Alegre, Brazil; 6 Endocrinology Service, Hospital de Clínicas de Porto Alegre, Porto Alegre, Brazil; 7 Hospital Israelita Albert Einstein, Goiânia, Brazil; 8 Hospital das Clínicas da Universidade Federal de Goiás, Goiânia, Brazil; 9 Endocrinology and Metabolism Service, Hospital BP-Beneficência Portuguesa of São Paulo, São Paulo, Brazil; 10 Instituto Do Coração, Hospital das Clínicas da Faculdade de Medicina da Universidade de São Paulo, São Paulo, Brazil; 11 Endocrinology Service, Hospital Santa Izabel da Santa Casa da Bahia, Salvador, Brazil; 12 Internal Medicine Department, State University of Rio de Janeiro, Rio de Janeiro, Brazil; 13 Nutrology and Diabetes Service, Federal University of Rio de Janeiro, Rio de Janeiro, Brazil; 14 Hospital Israelita Albert Einstein, São Paulo, Brazil; 15 Internal Medicine Department, Universidade Federal de Minas Gerais, Belo Horizonte, Brazil; 16 Endocrinology Service, Mater Dei Hospital, Belo Horizonte, Brazil; 17 Endocrinology and Metabolism Service, Federal University of Pernambuco, Recife, Brazil; 18 Hospital Clínica São Vicente, Rio de Janeiro, Brazil; 19 Present Address: Endocrinology and Metabolism Service, Hospital Nossa Senhora das Graças, Curitiba, Brazil

## Abstract

**Background:**

Hospital Hyperglycemia (HH) is linked to poorer outcomes, including higher mortality rates, increased ICU admissions, and extended hospital stays, and occurs in both people living with diabetes or not. The prevalence of HH in non-critical patients ranges from 22 to 46%. This panel reviewed the evidence and made recommendations for the best care for hospitalized hyperglycemic patients, with or without diabetes mellitus.

**Methods:**

The methodology was published previously and was defined by the internal institutional steering committee. The SBD Acute and Hospital Complications Department drafted the manuscript, selecting key clinical questions for a narrative review using MEDLINE via PubMed. The best available evidence was reviewed, including randomized clinical trials (RCTs), meta-analyses, and high-quality observational studies related to Hospital Hyperglycemia.

**Results and conclusions:**

The department members and external experts developed 23 recommendations for the management of patients with HH, including screening, initial interventions, treatment adjustments, and care for potential complications. Based on the best available evidence, our article provides safe and effective management strategies for both public and private healthcare settings.

## Introduction

Hospital Hyperglycemia (HH) is traditionally defined as capillary or plasma glucose levels above 140 mg/dL in hospitalized individuals [1–5]. However, pharmacological treatment is indicated for glucose levels exceeding 180 mg/dL, due to increased risks of complications and outcomes outside the targets for in-hospital glycemic control. HH affects individuals regardless of diabetes mellitus status, leading to worse outcomes such as higher hospital mortality rates, increased intensive care units (ICU) admissions, and longer hospital stays [1–5].

HH has been reported in 22–46% of non-critical hospitalized patients. Data from the Annual National Diabetes Inpatient Audit in the United Kingdom suggest that the prevalence of diabetes in hospitalized patients increased from 15% in 2010 to nearly 20% in 2019. Glycemic control in hospitalized patients often remains underestimated, contributing to clinical inertia [6].

Improved glycemic management has been shown to reduce complications like infections, particularly those related to surgical sites and pneumonia, and hospital costs, especially when conducted by specialized teams dedicated to in-hospital glycemic control [7–11].

Persistent HH, defined as glucose levels >180 mg/dL on two or more occasions within 24 h, require proactive glycemic control methods beyond sliding-scale insulin [1–3].

The treatment that is accessible in all hospitals, whether public or private, is insulin therapy. It should be prescribed with predetermined doses, as suggested below and in the recommendations (Table 1):

**Table 1:** Approach to glycemic control in various blood glucose ranges

Randomly discovered blood glucose in hospitalized patient	Action
Blood Glucose Level <140 mg/dL in Patients without Diabetes or Risk Factors for Stress Hyperglycemia (The specific risk factors are detailed in the footnote)	No need for follow-up
**140–180 mg/dL** or presence of Risk Factors* (The specific risk factors are detailed in the footnote)	• Capillary Blood Glucose Monitoring (CBGM) • Preprandial Correction Insulin for Occasional Hyperglycemia—Tables 2 and 4 (Less than one episode per day above 180 mg/dL and all below 250 mg/dL)
**180–200 mg/dL** AND **No outpatient use of insulin**	• CBGM • Basal Insulin Therapy Combined with Preprandial Corrections—Tables 2 and 4; or • DPP-4 Inhibitors Associated with Preprandial Corrections;
**200–250 mg/dL** or **Outpatient use of insulin with a total dose below 0.6 IU/kg**	• CBGM • Basal Insulin Therapy Combined with Preprandial Corrections—Tables Table 2 and 4
**>250 mg/dL,** or **History of type 1 diabetes, LADA, Secondary diabetes due to Pancreatectomy,** or **Outpatient use of Insulin with a total dose above 0.6 IU/kg**	• CBGM • Basal-Bolus Insulin Therapy
**Hyperglycemia Secondary to Glucocorticoids Use**	• Basal-Bolus Insulin Therapy with/or NPH Insulin in the Morning, Proportional to the Glucocorticoid Dose

^*^RISK FACTORS

Use of glucocorticoids, Post-organ transplantation, Postoperative period (24–48 h), Enteral or parenteral nutrition, Fasting state, Use of glucose-containing solutions, Systemic inflammatory response syndrome (SIRS), Sepsis, Arterial hypertension, Dyslipidemia, Obesity, Previous history of hospital hyperglycemia or diabetes mellitus

## Methodology

This review is an English-translated update of part of the 2024 SBD Guidelines, and the methodology was approved for publication by the internal institutional steering committee. In brief, the SBD appointed the experts of the central committee, which regulated the methodology, reviewed the manuscripts, and judged the degree of recommendations and level of evidence. The SBD Acute and Hospital Complications Department drafted the manuscript, selecting key clinical questions for a narrative review using MEDLINE via PubMed. The best available evidence was reviewed, including randomized clinical trials (RCTs), meta-analyses, and high-quality observational studies related to Hospital Hyperglycemia.

### Level of evidence

Three levels of evidence were considered: **A**—Data from more than one RCT or a meta-analysis of RCTs with low heterogeneity (I2 < 40%). **B**—Data from a meta-analysis with high levels of heterogeneity (I2 ≥ 40%), a single RCT, a prespecified subgroup analysis, large observational studies, or meta-analyses of observational studies. **C**—Data from small or nonrandomized studies, exploratory analyses, other guidelines, or expert consensuses.

### Degree of recommendation

For each defined recommendation, a poll was sent to all experts from the Acute and Hospital Complications Department and from the central committee. The frequency of the responses was analyzed, and a degree of recommendation was obtained based on the following criteria: **I**—More than 90% of the panel agreed; **IIa**—between 70 and 90% of the panel agreed; **IIb**—between 50–70% of the panel agreed; and **III**—Most of the panelists advised against a specific intervention. The terminology for the four degrees of recommendation was as follows: **I—IS RECOMMENDED**; **IIa—SHOULD BE CONSIDERED**; **IIb—MAY BE CONSIDERED**; and **III—IS NOT RECOMMENDED**.

## Recommendations

### Definition of hospital hyperglycemia

**Table:** 

**R1:** It is RECOMMENDED to use the criterion of HOSPITAL HYPERGLYCEMIA for all individuals with capillary or plasma glucose levels above 140 mg/dL, regardless of the prior existence of diabetes, as it correlated with worse outcomes	
I	B

#### Summary of evidence

- Murad MA et al. published a meta-analysis with a systematic review of the literature in 2012, with the objective of evaluating the impact of glycemic control on the clinical outcomes of non-critical patients. The analysis included nine randomized studies and 10 observational studies, with goals Intensive group glycemic control ranging from 100 to 180 mg/dL A significant reduction in the relative risk of infection was observed with intensive glycemic control (RR = 0.41, 95% CI 0.21–0.77) [4].
- McAlister F et al., in a multicenter prospective study involving 2,471 patients hospitalized for community-acquired pneumonia, 44% of whom had diabetes, reported that patients with admission glucose levels >198 mg/dL had higher mortality (13 vs 9%, *p* = 0.03) and hospital complications (29 vs 22%, *p* = 0.01) [5].
- Bhatti JM et al. evaluated the association between glycemic control and clinical outcomes in hospitalized COVID-19 patients (n = 638). They observed a higher risk of mechanical ventilation with admission glucose levels above 250 mg/dL and a greater need for intensive care support with admission glucose levels above 160 mg/dL [6].
- Van den Boom et al., in a retrospective analysis of 431,480 surgeries at Duke University Health System, highlighted the association between the average capillary glucose levels in the first three days postoperatively and 30-day mortality. For cardiac procedures, there was a U-shaped relationship between glucose and mortality, with mortality rates ranging from 4.5% at 100 mg/dL to a nadir of 1.5% at 140 mg/dL, rising again to 6.9% at 200 mg/dL. Due to the increased complications with glucose levels above 140 mg/dL, this cutoff has been proposed as a marker by some authors [7].
- Kosiborod et al., in a retrospective study of 16,871 acute myocardial infarction cases in patients with and without diabetes, evaluated average glucose levels at 24, 48, and 72 h, as well as throughout the hospital stay. They observed consistent increases in mortality above 70 mg/dL and for every 10 mg/dL above 120 mg/dL, with statistically significant differences when comparing values of 140–170 mg/dL to the reference range below 110 mg/dL and values of 110–140 mg/dL [8].
- Fong et al., in a retrospective study of 52,107 ICU patients across 208 US hospitals, including 15,652 diagnosed with diabetes, evaluated average glucose levels (AG), episodes of hypoglycemia, and glycemic variability by coefficient of variation (CV) related to mortality in groups with and without diabetes. For patients without diabetes, there was a J-shaped association between AG and mortality, with the lowest mortality risk in the 80–120 mg/dL range. In contrast, for patients with diabetes there was a right-shifted and attenuated association between AG and hospital mortality, with the lowest mortality risk in the 90–150 mg/dL range. Hypoglycemia was associated with increased mortality in both groups, but to a lesser degree in patients with diabetes. An association between CV and hospital mortality was observed only in patients without diabetes [9].

**Table:** 

IMPORTANT NOTE 1: Persistent Hospital Hyperglycemia
• The term “Persistent Hospital Hyperglycemia” should be applied to all patients who exhibit two or more episodes of capillary or plasma glucose levels above 180 mg/dL within 24 h, as these levels are associated with worse clinical outcomes and a greater need for treatment
• In the presence of persistent hospital hyperglycemia, proactive therapeutic intervention for glycemic control is necessary, which may include basal insulin combined with supplemental preprandial insulin correction, basal-bolus insulin therapy, or the use of oral antidiabetic agents (as specified in the recommendations below)

### Screening: when to measure blood glucose in hospitalized patients (Fig. 1)

**Table:** 

**R2:** It is RECOMMENDED to screen for hospital hyperglycemia with a capillary or plasma glucose test in **all adult inpatients** upon admission, regardless of prior diabetes diagnosis	
I	C

#### Summary of evidence

- Abdelmalak et al. conducted a study evaluating the prevalence of undiagnosed diabetes in patients undergoing non-cardiac surgery and found a prevalence of 10%. Additionally, they discovered that the average preoperative blood glucose concentration in patients with undiagnosed diabetes was higher than in those who were aware of their diabetes diagnosis [10].
- Considering that many people in Brazil with diabetes are unaware of their diagnosis, it is rational to screen for hospital hyperglycemia in patients without a known diabetes diagnosis [11].
- Umpierrez et al., in a retrospective study of 2,030 consecutive hospitalizations, found that 38% of admissions to a general hospital involved hospital hyperglycemia, defined as blood glucose greater than 126 mg/dL. Of these cases, 12% had no prior diabetes diagnosis, while 26% had a known diagnosis. Mortality was higher in patients without a prior diabetes diagnosis (16%) compared to those with diabetes (3%) and those without hyperglycemia (1.7%) (*p* < 0.01 for both), highlighting both the prevalence and increased severity of hyperglycemia in these patients [12].

### When to measure HbA1c in hospitalized patients (Fig. 1)

**Table:** 

**R3**: It is RECOMMENDED to measure HbA1c in all patients with confirmed hospital hyperglycemia or pre-existing diabetes, provided testing has not been done in the past three months. This assists in diagnosing previously undetected diabetes when HbA1c is above 6.5%, and supports discharge planning	
I	**B**

#### Summary of evidence

**Fig. 1:**
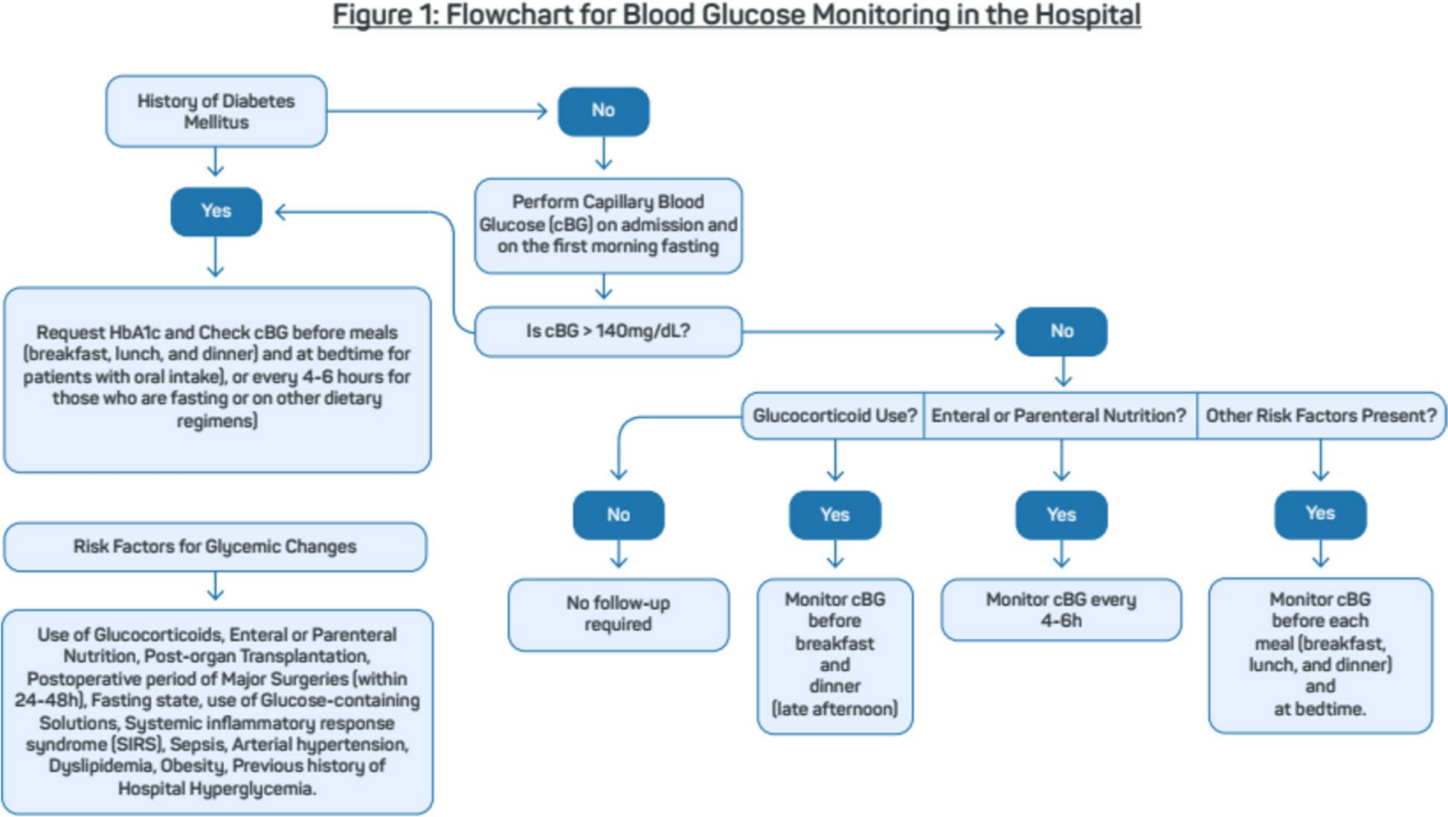
Flowchart for Blood Glucose Monitoring in the Hospital

- Measuring HbA1c in hospitalized patients allows for the differential diagnosis between diabetes and stress-induced hyperglycemia. Evaluating glycemic control prior to admission can predict the occurrence of dysglycemia during hospitalization, aiding in therapeutic optimization.
- In hospitalized patients with hyperglycemia, diabetes can be diagnosed with an HbA1c level ≥6.5%, in the absence of interfering factors (see important note 5, as recommended in the “Diagnosis of diabetes and screening for type 2 diabetes” chapter of the SBD Guidelines) [11].
- Greci et al. evaluated the usefulness of HbA1c in diagnosing new cases of diabetes in hospitalized patients with hyperglycemia. An HbA1c level >6.0% had a sensitivity of 57% and a specificity of 100% for diagnosing diabetes, while an HbA1c level <5.7% excluded the diagnosis of diabetes with a sensitivity of 100% and a specificity of 50% [13].
- Pasquel et al. assessed the role of admission HbA1c in predicting glycemic control during hospitalization in a systematic review compiling data from four controlled and randomized studies. Patients with elevated HbA1c levels had a lower chance of achieving good glycemic control during hospitalization compared to those with HbA1c ≤7% (HbA1c >7–9%, odds ratio 0.45 [95% CI 0.22–0.92]; HbA1C >9%, 0.37 [95% CI 0.17–0.75]). Patients with HbA1c ≤7% required lower doses of insulin during hospitalization [14] (Fig. 1).

**Table:** 

IMPORTANT NOTE 2: Interferents in HbA1c Measurement
• The laboratory analysis of HbA1c was standardized by the high-performance liquid chromatography (HPLC) method, and its validation needs to be certified by the National Glycohemoglobin Standardization Program (NGSP), established for applicability in the Diabetes Control and Complications Trial (DCCT) study [15]
• Conditions affecting the red blood cell life cycle, such as anemia and hemolysis, drugs that increase erythropoiesis, chronic kidney disease, pregnancy, and hemoglobinopathies, can cause discrepancies between the HbA1c value and the estimated average glucose [16]
• Fructosamine may be an alternative for assessing glycemic control prior to hospitalization in patients with interferents affecting HbA1c evaluation; however, the assays are not standardized, making it difficult to establish a cutoff value for diagnosis [17–19]

### Monitoring glycemia in non-critical patientS

**Table:** 

**R4**: It is RECOMMENDED to monitor capillary glucose levels in all patients with hospital hyperglycemia, diabetes, or risk factors	
I	C

#### Summary of evidence

- All randomized studies used fasting and pre-meal blood glucose levels as the basis for titrating insulin or oral antidiabetic drugs (OADs). Therefore, measuring blood glucose at these times is extremely important, despite the lack of studies comparing the best frequency for testing in patients with hospital hyperglycemia.
- Vellanki et al. evaluated the relevance of bedtime glucose monitoring and its role in managing glycemia. The study found no significant improvement in fasting blood glucose levels or overall glycemic control from the use of bedtime insulin supplementation in patients with type 2 diabetes, challenging current guidelines recommending routine bedtime supplementation for hyperglycemia correction. Hypoglycemia rates did not differ significantly between groups that received or did not receive bedtime insulin supplementation. Importantly, the majority of hypoglycemic episodes occurred between dinner and early morning, emphasizing the utility of bedtime glucose monitoring in identifying at-risk patients [20].
- Bedtime blood glucose is an important safety parameter to avoid nighttime hypoglycemia in insulin-treated patients. It allows for checking the rise or fall of glucose levels during the night and helps in adjusting basal insulin doses [1, 2, 21, 22].
- Ribeiro et al. evaluated the impact of introducing an institutional glucose screening and monitoring program in a private Brazilian hospital, increasing monitoring from 27.7 to 75.5% of inpatients. This led to an increase in the identification of hyperglycemia from 9.3 to 12.2% and hypoglycemia from 1.5 to 3.3% of inpatients [23].

**Table:** 

IMPORTANT NOTE 3: WHEN TO MONITOR BLOOD GLUCOSE IN THE HOSPITAL
• Capillary blood glucose monitoring should be individualized according to each patient’s profile: ○ Before oral diets ○ Every 4–6 h for continuous diets ○ At 3 a.m. if there is a risk of nighttime hypoglycemia ○ For pregnant women, 1 h after meals
• It is important to measure venous or plasma glucose when the capillary glucose value is above the upper detection limit of the glucometer (HI Alert)
• Check the device’s instructions to know the accepted glycemia limit. If the reading is above this limit, it is crucial to perform a plasma or arterial test to accurately determine the patient’s glucose level, enabling correct diagnosis and monitoring of treatment progress

**Table:** 

IMPORTANT NOTE 4: HOSPITAL-USE GLUCOMETERS
• Hospitals should use validated devices for hospital use, with frequent accuracy evaluations to ensure results are compatible with plasma glucose values, making capillary blood glucose values equivalent to plasma glucose
• These glucometers integrate with most hospital systems, ensuring results are linked to respective patients and recorded in a standardized location. This facilitates blood glucose control and allows active case tracking for those not being monitored
• With this data, it is possible to trace and identify the entire process for failures and improvements, ensuring the results are accurate. Using home-use glucometers for this purpose requires significant commitment from the entire care team, with precise recording in designated fields for capillary glucose in medical records, frequent comparisons with simultaneous plasma samples, and consideration of the interfering factors mentioned in IMPORTANT NOTE 5 [24]

**Table:** 

IMPORTANT NOTE 5: INTERFERENCES IN CAPILLARY BLOOD GLUCOSE
1. Training and competence of the staff
2. Compromised peripheral circulation
3. Extremes of hematocrit
4. Hypoxia
5. Acidosis or alkalosis
6. Interference from other substances (acetaminophen, vitamin C, uric acid, triglycerides, icodextrin)
7. Alterations in protein and lipid levels
8. Typing errors by the healthcare team
9. Inadequate hand hygiene

**Table:** 

**R5:** It is RECOMMENDED to test for capillary ketonemia or ketonuria in the following situations: blood glucose levels above 200 mg/dL and signs and symptoms suggestive of ketoacidosis; or patients on SGLT2 inhibitors with signs and symptoms of ketoacidosis, even with normal blood glucose levels (euglycemic diabetic ketoacidosis)	
I	C

#### Summary of evidence

- Capillary ketonemia measurement is an accurate and quick method for detecting serum ketone bodies. The test for detecting ketones in capillary blood has a sensitivity of 98% and a specificity of 79%, compared to the urine ketone test, which has a sensitivity of 98% and a specificity of 35% [25].
- A systematic review of 5 studies, including 2019 patients, identified greater accuracy in measuring beta-hydroxybutyrate in capillary blood compared to ketonuria for diagnosing diabetic ketoacidosis (DKA) [26].
- Euglycemic DKA (defined as glucose levels below 200 mg/dL) is a potential complication in patients using SGLT2 inhibitors and is underdiagnosed in the hospital setting [27].
- In Canada and the UK (over 350,000 patients and 500 events of euglycemic DKA), SGLT2 inhibitors were associated with a higher risk of DKA compared to DPP-4 inhibitors with incidence 2.03 vs. 0.75 per 1000 patient-years (RR = 2.85, 95% CI 1.99–4.08) [28].

**Table:** 

**R6:** In patients using glucocorticoids, regardless of a prior diagnosis of diabetes mellitus, IT IS RECOMMENDED to perform capillary glucose measurements upon waking and before dinner	
I	C

#### Summary of evidence

- Toyoshima et al., in an observational pilot study, prospectively monitored 15 patients undergoing chemotherapy for hematological malignancies, using dexamethasone or prednisone using continuous glucose monitoring (CGM) sensors. They found hyperglycemia in 60% of the sample, with notable rises in the afternoon and early evening, particularly when administered in the morning [29].
- Donihi et al., in a retrospective study involving 50 patients receiving the equivalent of 40 mg of prednisone daily, observed hyperglycemia in 64% of all patients, including 52% among those without a prior diagnosis of diabetes mellitus [30].
- Le et al., in a retrospective observational study involving 517 patients admitted with severe COVID-19, reported a 65% incidence of hospital-acquired hyperglycemia, particularly prevalent in patients with respiratory failure and receiving glucocorticoids, consistent with other publications [31].
- The Endocrine Society Guidelines (2012) recommend that glucose monitoring may be discontinued in patients receiving glucocorticoid therapy if, during a 24–48 h monitoring period, no glucose readings exceed 140 mg/dL [32]. However, this recommendation is based on expert consensus, and no specific studies have validated the safety of this timeframe. The absence of empirical evidence necessitates cautious application of these guidelines, particularly in patients receiving high doses of long-acting glucocorticoids, such as dexamethasone, where the risk of prolonged hyperglycemia is greater.

### Glycemic targets for non-critical inpatients

**Table:** 

**R7:** In non-critical patients with or without diabetes, it is RECOMMENDED to aim for glycemic control targets between 100 to 180 mg/dL to avoid hyper- and hypoglycemia	
I	**B**

#### Summary of evidence

##### Historical context and pre-2009 evidence

- Before 2009, studies such as the RABBIT-2 (2007) and RABBIT-2 Surgery (2011) trials demonstrated clinical benefits of achieving glycemic targets between 100–140 mg/dL in hospitalized patients. The RABBIT-2 trial showed that a basal-bolus insulin regimen improved glycemic control compared to sliding-scale insulin (SSI) in non-critical patients with type 2 diabetes, achieving mean glucose levels of 166 versus 193 mg/dL [1]. Similarly, the RABBIT-2 Surgery trial demonstrated that basal-bolus insulin reduced perioperative complications, such as wound infections and pneumonia, compared to SSI [2].
- McAlister et al., in a multicenter prospective study involving 2471 patients hospitalized for community-acquired pneumonia (44% of whom had diabetes), reported that patients with admission glucose levels >198 mg/dL, compared to those with lower glucose levels, had higher mortality (13 vs. 9%, *p* = 0.03) and hospital complications (29 vs. 22%, *p* = 0.01) [5].
- Kosiborod et al., in a retrospective study of 16,871 cases of acute myocardial infarction in patients with or without diabetes mellitus, average glucose levels were assessed at 24, 48, and 72 h, as well as throughout the entire hospitalization. Consistent increases in mortality were observed with each 10 mg/dL rise above 120 mg/dL, while worse outcomes were noted with glucose levels below 70 mg/dL (OR 6.4; *p* = 0.01). It is important to avoid both hypoglycemia and hyperglycemia in hospitalized patients [8].

##### NICE-SUGAR study and 2009 guideline changes

- The NICE-SUGAR study, conducted in critically ill patients, revealed that tight glycemic control (81–108 mg/dL) was associated with increased mortality compared to a more moderate glycemic target of 140–180 mg/dL [33]. These findings led to changes in the glycemic target for critically ill patients in the 2009 Consensus Statement on Inpatient Glycemic Control by the American Association of Clinical Endocrinologists (AACE) and American Diabetes Association (ADA) [34]. Although the NICE-SUGAR study was designed for critically ill patients, its results prompted the extrapolation of a similar target range (140–180 mg/dL) to non-critical hospitalized patients to balance the benefits of glycemic control with the risks of hypoglycemia.
- Since 2012, the Endocrine Society has recommended a glycemic target range of 100–180 mg/dL for non-critical hospitalized patients. This target aims to balance glycemic control while minimizing the risk of hypoglycemia, particularly in patients with diabetes or stress-induced hyperglycemia [32].
- Murad MA et al. published a meta-analysis with a systematic review of the literature in 2012, aiming to evaluate the impact of glycemic control on the clinical outcomes of non-critical patients. The analysis included nine randomized studies and 10 observational studies, with intensive group glucose targets ranging from 100 to 180 mg/dL. A significant reduction in the RR of infection was observed with intensive glycemic control (RR = 0.41, 95% CI 0.21–0.77) [4].
- Fong et al., in a retrospective study of 52,107 patients admitted to ICU in 208 North American hospitals, including 15,652 patients with diabetes, assessed average glucose levels (AGL), hypoglycemia, and glycemic variability by coefficient of variation related to mortality in groups with and without diabetes. Patients without diabetes showed a J-shaped association between AGL and mortality, with a lower mortality risk at an average glucose range of 80–120 mg/dL. In contrast, patients with diabetes showed a right-shifted attenuated association between AGL and hospital mortality, with a lower mortality risk at an average glucose range of 90–150 mg/dL. Hypoglycemia was associated with increased mortality in both groups but to a lesser degree in patients with diabetes. An AGL >180 mg/dL was associated with a relative mortality risk of 4.20 (4.07–4.33) in patients without diabetes and 1.14 (1.08–1.20) in patients with diabetes, using an AGL of 100 mg/dL as a reference and adjusting for other risk factors. The association between CV (coefficient of variation) and hospital mortality was observed only in patients without diabetes [9].
- Bhatti JM et al. evaluated the association between glycemic control and clinical outcomes in hospitalized COVID-19 patients (n = 638), observing a higher risk of mechanical ventilation with admission glucose levels above 250 mg/dL and a greater need for intensive care support with admission glucose levels above 160 mg/dL [6].
- The randomized clinical trial SHINE included 1151 patients with acute stroke and reported no benefits in functional outcomes with intensive glycemic control (glucose targets 80–130 mg/dL) compared to standard glycemic control (glucose targets 80–179 mg/dL) [35].

##### 2022 American diabetes association updates

- In 2023, the American Diabetes Association updated its standards of care to align with the Endocrine Society’s recommendations, adopting a glycemic target range of 100–180 mg/dL for non-critical hospitalized patients [36]. This change reflects evolving evidence and a broader consensus on optimal glycemic management in this patient population.

### Treatment of hospital hyperglycemia (Tables 2, 3, 4) (Fig. 2)

**Table:** 

**R8:** Scheduled basal insulin combined with pre-prandial bolus IS RECOMMENDED for treating persistent hyperglycemia in non-critical hospitalized patients. This approach is associated with better glycemic control, reduction of adverse outcomes, and shorter hospital stays	
I	**A**

**Table:** 

IMPORTANT NOTE 6: Insulin Therapy Regimens in the Hospital
Hospital insulin therapy can be administered in the following ways:
1. **Preprandial Correction insulin only, based on preprandial glucose levels:** Occasional and non-persistent hyperglycemia can be corrected with short-acting insulin or rapid-acting or ultra-rapid insulin analogs, using a correction table as suggested in Table 4 or one standardized by the institution. This may also complement the dose already prescribed in a basal-plus or a basal-bolus therapy
2. **Basal-plus** a. Long-acting or intermediate-acting) combined with preprandial correction insulin
3. **Basal-bolus therapy:** Basal insulin is associated with preprandial boluses of short-acting insulin or rapid-acting or ultra-rapid insulin analogs before meals in patients on oral diets, or every 4–6 h in patients on continuous enteral or parenteral feeding. These doses are predefined and supplemented with preprandial correction insulin

#### Summary of evidence

##### Sliding scale insulin

- All studies comparing the sliding scale insulin (SSI) regimen with other regimens have shown the inferiority and ineffectiveness of the sliding scale in controlling hyperglycemia. Therefore, when hospitalized patients present with more than two blood glucose levels above 180 mg/dL in 24 h, any hyperglycemia above 250 mg/dL, or are already using insulin at home, an effective hyperglycemia treatment should be implemented. Using the sliding scale alone is contraindicated. There are no studies comparing the efficacy of different types of correction scales. Table 4 presents the authors’ suggestion for a supplemental insulin correction table. The chosen scale should be standardized by the institution and adjusted to effectively lower blood glucose levels without causing hypercorrection.

##### Basal-plus insulin therapy

- Umpierrez et al. conducted a multicenter randomized study with 375 patients divided into three groups: basal-bolus, basal combined with correction scale (referred to as basal-plus in the study), and SSI alone. The study included hospitalized patients with type 2 diabetes mellitus, using oral medications or insulin up to a dose of 0.4 IU/kg/day. The basal-bolus and basal-plus groups achieved similar results, with less hyperglycemia and lower average daily blood glucose levels than the SSI group, without an increase in severe hypoglycemia. However, both basal groups experienced an increase in mild hypoglycemia compared to the SSI group [37].
- Pasquel et al. evaluated a group of 279 hospitalized patients with type 2 diabetes, using oral medications or insulin up to a total dose of 0.6 IU/kg/day and blood glucose levels below 400 mg/dL, in a multicenter randomized study. They compared a group receiving basal insulin combined with sitagliptin to a group on basal-bolus insulin therapy. The outcomes were not inferior in terms of average daily blood glucose levels (171 ± 48.6 vs. 169.2 ± 48.6 mg/dL), hypoglycemia (9 vs. 12%), and treatment failure (16 vs. 19%) for the sitagliptin + basal group vs. the basal-bolus group, respectively [21].

##### Basal-bolus insulin therapy

- Umpierrez et al. compared the safety and efficacy of the basal-bolus regimen with once-daily glargine and pre-meal glulisine to a SSI regimen in a multicenter study involving 213 type 2 diabetes patients undergoing surgery. The mean glucose level after the first day of the basal-bolus regimen was 145 ± 32 mg/dL, compared to 172 ± 47 mg/dL in the SSI group (*p* < 0.01). Glucose levels below 140 mg/dL occurred in 55% of patients in the basal-bolus group and only 31% in the SSI group (*p* < 0.001). There was a 64.6% reduction in the composite endpoint of mortality, surgical site infection, pneumonia, and acute renal failure in the basal-bolus group, mainly due to reduced surgical site infection and acute renal failure [2].
- In another multicenter study by Umpierrez et al. involving 130 non-surgical, insulin-naive type 2 diabetes inpatients, the safety and efficacy of the basal-bolus regimen with once-daily glargine and pre-meal glulisine were compared to a SSI regimen. The basal-bolus group achieved average daily glucose levels that were 27 mg/dL lower, without an increase in hypoglycemia. Despite increasing doses in the SSI group, 14% of patients remained with glucose levels above 240 mg/dL [1].
- Christensen MB et al. conducted a systematic review and meta-analysis to evaluate the safety and efficacy of the basal-bolus regimen in hospitalized type 2 diabetes patients. Five clinical trials and seven observational studies were included. The meta-analyses demonstrated an average daily glucose level 14–29 mg/dL lower with the basal-bolus regimen compared to the SSI regimen. The risk of hypoglycemia was higher in the basal-bolus group compared to the SSI group (glucose ≤70 mg/dL, RR 5.75; 95% CI 2.79–11.83), (glucose ≤60 mg/dL, RR 4.21; 95% CI 1.61–11.02). There was no difference in the rates of hypoglycemia below 40 mg/dL between the groups [3].
- In their study comparing basal-bolus and basal-plus insulin therapy regimens, Umpierrez et al. replaced the basal-plus regimen with the basal-bolus regimen when the average daily glucose levels or two consecutive glucose readings were greater than 240 mg/dL [37].
- Bellido et al. in a prospective, open-label randomized trial, comparing an intermediate-acting insulin (NPH) regular insulin 70/30 mixture with a basal-bolus regimen in hospitalized patients found comparable glycemic outcomes but a significantly higher incidence of hypoglycemia in the group receiving the insulin mixture. Therefore, the use of pre-mixed insulins should be restricted to individual cases with previous use, not being used as standard [38].
- There are no studies evaluating the transition between insulin therapy regimens. However, when the method used is not effective, it is recommended to switch to a more effective regimen, which in this case would be the basal-bolus regimen.
- None of the above studies randomized patients with insulin doses above 0.6 IU/kg/day to arms not using the basal-bolus therapy (Tables 2, 3, 4).

**Table 2:** Choice of insulin regimen in the hospital for non-critical inpatients

Insulin regimen	Indication	Level of Recommendation and Evidence	
**Sliding Scale** (Only ultra-rapid, rapid or short preprandial insulin)	• Maximum of one blood glucose >180 mg/dL per day and no blood glucose above 250 mg/dL; No outpatient use of insulin	**IIb**	**B**
**Basal-plus** (Basal insulin + correction scale)	• 180–250 mg/dL; • Outpatient insulin dose <0.6 IU/kg/day	**IIa**	**B**
**Basal-bolus** (Basal insulin + multiple doses of bolus insulin + correction scale)	• >250 mg/dL; • Type 1 Diabetes, LADA, Diabetes secondary to pancreatectomy; • Outpatient insulin dose >0.6 IU/kg/day	**I**	**B**

**Table 3:** Calculation of the basal-bolus insulin regimen dose

**Basal Insulin**
Establish the Total Daily Dose (TDD) of insulin (0.2–0.6 UI/kg/day)
Basal dose = 50% of TDD or 0.1–0.3 UI/kg/day
**Types of Basal Insulin:**
• NPH: 2–3 doses per day (For Type 1 diabetes, LADA, or pancreatectomized patients, prefer 3 times a day)
• Glargine U100: 1 or 2 doses per day
• Degludec and Glargine U300: 1 dose per day (These can be used, but attention should be given to their longer half-life and the 3–4 day period required to reach steady state, which may complicate management in hospitalizations or short-term follow-ups)
**Bolus Insulin**
**Bolus dose = 50% of TDD**
• If on oral or intermittent enteral diet:
1/3 of the bolus dose per meal (50% TDD/3 before breakfast, lunch, and dinner)
• If on continuous enteral/parenteral diet:
¼ of the bolus dose every 6 h
• If fasting:
Do not use
**Types of Insulin for Bolus:**
• Rapid-acting or ultra-rapid analogs: (Lispro, Glulisine, Aspart, Fast-Acting Aspart)
Should be administered up to 15 min before meals
• Short-acting insulin: Regular human insulin:
Should be administered 30 min before meals

**Table 4:** Suggested blood glucose correction scale insulin*

Capillary blood glucose	Sensitive^1^	Usual^2^	Resistant^3^
141–180 mg/dL	No Supplemental Dose	Increase by 1 IU	Increase by 2 IU
181–220 mg/dL	Increase by 1 IU	Increase by 2 IU	Increase by 4 IU
221–260 mg/dL	Increase by 1 IU	Increase by 3 IU	Increase by 6 IU
261–300 mg/dL	Increase by 2 IU	Increase by 4 IU	Increase by 8 IU
301–340 mg/dL	Increase by 2 IU	Increase by 5 IU	Increase by 10 IU
341–380 mg/dL	Increase by 3 IU	Increase by 6 IU	Increase by 12 IU
381–420 mg/dL	Increase by 3 IU	Increase by 7 IU	Increase by 14 IU
>420 mg/dL	Increase by 4 IU	Increase by 8 IU	Increase by 16 IU

*CF* correction factor, *GTR* blood glucose target range

^*^Short-acting insulin or rapid-acting or ultra-rapid analogs. In the case of continuous enteral or parenteral feeding, correction can be performed every 4–6 h

(1) **Sensitive** (CF 80 mg/dL and GTR 100–140 mg/dL): indicated for elderly patients, or with a body mass index (BMI) < 19 kg/m^2^, frail and/or with kidney, liver or heart failure

(2) **Usual** (CF 40 mg/dL and GTR 100–140 mg/dL): indicated for patients with BMI between 19 and 33 kg/m^2^, without signs of insulin resistance or use of glucocorticoids

(3) **Resistant** (CF 20 mg/dL and GTR 100–140 mg/dL): indicated for patients with BMI above 33 kg/m^2^, with signs of insulin resistance, using glucocorticoids, or persistently elevated blood glucose levels

**Fig. 2:**
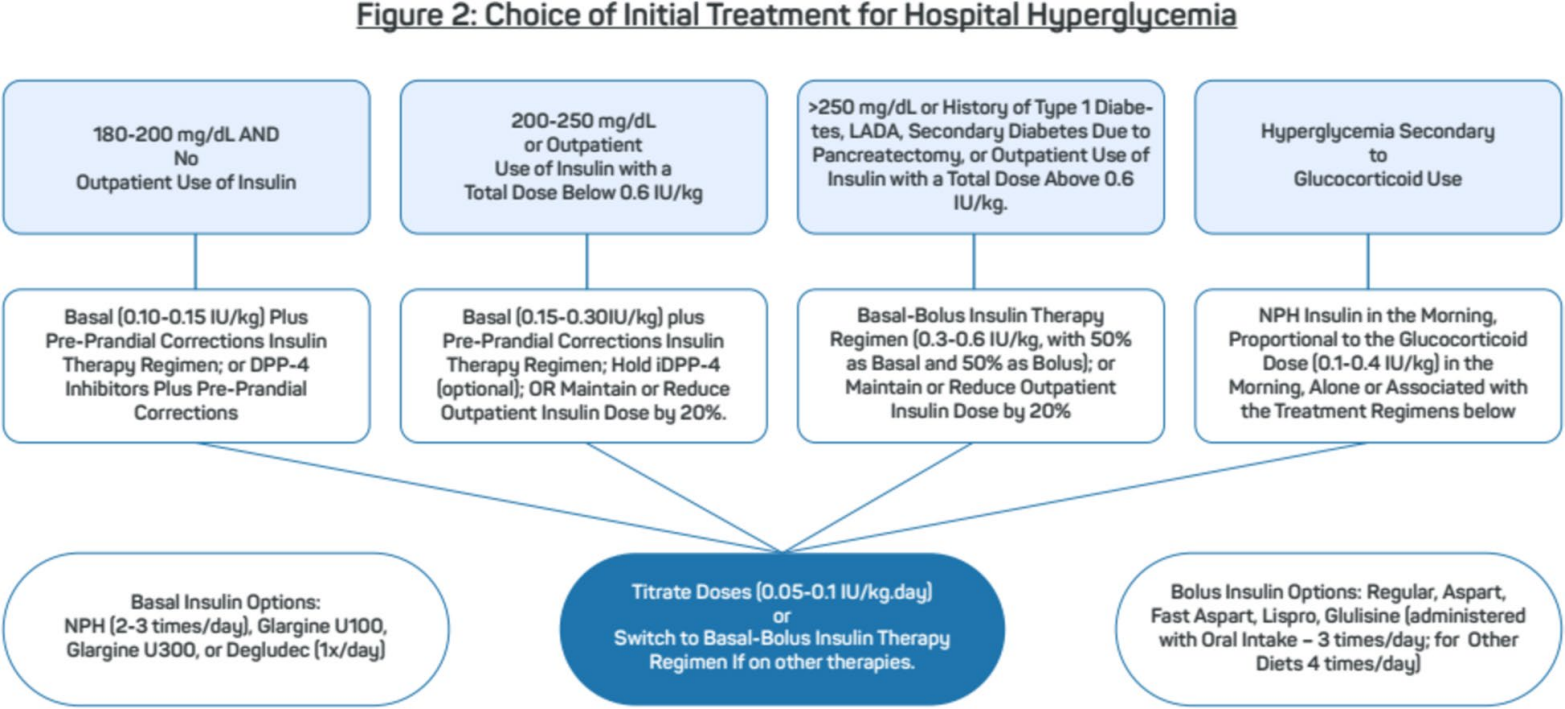
Choice of initial treatment for hospital hyperglycemia

**Table:** 

IMPORTANT NOTE 8: Insulin Therapy in Enteral and Parenteral Nutrition
• The prescription of basal insulin can be done with a once-daily long-acting analog insulin or NPH insulin two to three times a day, corresponding to 50–80% of the daily dose
• Bolus insulin with correction can be administered every 4–6 h with rapid-acting analogs or every 6 h with regular insulin, corresponding to 20–50% of the total daily dose
• In the case of enteral nutrition, it is important to consider whether the nutrition is continuous or intermittent and to coordinate the administration of rapid-acting insulin with feeding periods, preferring diets with a lower glycemic index
• It is crucial to monitor interruptions in these diets, and whenever basal insulin is used subcutaneously, upon diet interruption, intravenous glucose should be administered to minimize the risk of hypoglycemia [39]

**Table:** 

**R9:** IT MAY BE CONSIDERED to use a single daily dose of NPH insulin in the morning for the treatment of hyperglycemia secondary to glucocorticoid use in patients with predominantly afternoon hyperglycemia, starting at 0.1 IU/kg/day, either alone or in combination with other forms of insulinization or oral medications	
IIb	B

#### Summary of evidence

- Brooks et al., in a systematic review, demonstrated that the addition of intermediate-acting insulin (NPH insulin) to the conventional hypoglycemic regimen was safe and effective in controlling blood glucose secondary to glucocorticoid use in a hospital setting [40].
- Khowaja et al. conducted a randomized study (n = 61) showing that the addition of NPH insulin in the morning to the previous insulin regimen resulted in lower average daily blood glucose, as well as lower fasting and pre-meal blood glucose values in patients with diabetes using glucocorticoids during hospitalization [41].
- Grommesh et al., in a pilot randomized study with 61 patients using NPH insulin administered at the time of glucocorticoid use, showed superiority in glycemic control for the regimen combining NPH insulin with the basal-bolus scheme compared to long-acting basal insulin or basal-bolus insulin alone [42]

**Table:** 

IMPORTANT NOTE 9: Hyperglycemia Secondary to Glucocorticoid Use
• Hyperglycemia secondary to glucocorticoid use typically occurs more intensely in the afternoon, with lower blood glucose levels in the morning, creating a hyperglycemia curve similar to the pharmacokinetic curve of NPH insulin [29]
• An insulin adjustment regimen based on experience from a single service was proposed according to the glucocorticoid dose. The regimen involves initiating NPH insulin at a dose of 0.1 IU/kg for every 10 mg of prednisone-equivalent dose, with increments of 0.1 IU/kg for each additional 10 mg, up to a maximum dose of 0.4 IU/kg. For example, 0.2 IU/kg for a 20 mg prednisone-equivalent dose, 0.3 IU/kg for 30 mg, and 0.4 IU/kg/day for doses of 40 mg or higher [43, 44]
• The insulin dose should be titrated based on morning fasting blood glucose and preprandial levels, especially before dinner, with increases of 10–20% of the total dose or 0.1 IU/kg/day, depending on the degree of hyperglycemia. Adjustments can be made 24 h after the adjusted dose is administered
• Considering the pharmacokinetic differences of different glucocorticoids, as well as interindividual differences, different patterns of blood glucose alterations may occur in patients, necessitating different insulin regimens. The use of NPH insulin in the manner suggested above applies to patients who predominantly or exclusively exhibit hyperglycemia in the afternoon [45]

### Insulin therapy adjustment

**Table:** 

**R10:** It is RECOMMENDED to adjust insulin therapy every 24–48 h, according to blood glucose monitoring, dietary status, and the dosage of medications with hyperglycemic effects	
I	C

#### Summary of evidence

- Magaji and Johnston, in a narrative review and expert opinion article, suggest that insulin therapy adjustments be made every 24–48 h [46].
- The research group at Emory University, Atlanta, USA, conducted the RABBIT-2, DEAN, and Basal-Plus studies, making insulin therapy adjustments every 24 h. Similarly, studies with ultra-long-acting insulin analogs (glargine U300 and degludec) in a hospital setting proposed dose adjustments every 24 h [47, 48].

**Table:** 

Important Note 10: Insulin Dose Adjustments in Non-Critical Hospitalized Patients
• Insulin doses may need to be adjusted frequently during a patient’s hospitalization, depending on capillary blood glucose results, medications in use, and caloric intake [46]
• Throughout the patient’s hospitalization, the titration of the daily total dose (DTD) of insulin in the basal-bolus regimen, i.e., increasing or decreasing the insulin dose during follow-up, can be done in two ways: [2, 49, 50] o In percentage values of the daily total dose (10–20%); o According to the daily total dose of insulin per kg of the patient’s weight (approximately 0.05–0.1 IU/kg/day)

**Table:** 

**R11:** It is RECOMMENDED that in a basal-bolus regimen, the adjustment of the total daily insulin dose be based on the average blood glucose level or fasting and pre-meal blood glucose values. In cases where individualization of basal or prandial doses per meal is required, it is SUGGESTED to consult a team specialized in glycemic control (e.g., endocrinologists or diabetologists)	
I	**B**

#### Summary of evidence

- Magaji and Johnston published a review and expert opinion article suggesting that fasting blood glucose is the best indicator of the adequacy of the basal insulin dose with insulin glargine U100 in the morning. The other blood glucose levels throughout the day reflect the adequacy of the rapid-acting insulin bolus doses with meals. Blood glucose levels before lunch reflect the adequacy of the breakfast dose, blood glucose before dinner reflects the lunch insulin dose, and bedtime blood glucose reflects the dinner rapid-acting insulin dose [46].
- Zhang et al. also used fasting blood glucose to adjust the insulin glargine U100 dose in a randomized clinical trial [49].
- In the DEAN study, a randomized clinical trial conducted in 2009, Umpierrez et al. recommended daily increases in basal insulin doses (detemir and NPH) if fasting and pre-dinner blood glucose levels were higher than 140 mg/dL. If fasting and pre-dinner blood glucose levels were below 100 mg/dL, basal insulin doses were reduced [51].
- Umpierrez et al., in their RABBIT-2 and Basal-Plus studies, randomized clinical trials comparing correction scale, basal-bolus, or basal-plus insulin therapy regimens using insulin glargine U100 or NPH as basal insulin, adjusted insulin therapy according to the average blood glucose since the last evaluation [1, 37].
- Pasquel et al., in a prospective randomized study comparing the use of glargine U300 and glargine U100 insulin in a hospital setting, adjusted insulin doses daily according to fasting and pre-dinner blood glucose levels [47].
- Galindo et al., in a prospective randomized study comparing the use of degludec and glargine U100 insulin in a hospital setting, adjusted insulin doses daily according to fasting blood glucose and pre-meal blood glucose levels [48].

**Table:** 

**R12:** It is RECOMMENDED to adopt a standardized approach for adjusting insulin doses, which can be equally effective using percentage values of the total daily dose (TDD) of insulin (10–20% per day) or according to the TDD of insulin per patient weight (0.05–0.1 IU/kg/day)	
I	**B**

#### Summary of evidence

- Umpierrez et al., in the randomized clinical trial RABBIT-2, compared basal-bolus insulin therapy with sliding scale insulin therapy. They proposed an insulin adjustment protocol, increasing the basal insulin dose (glargine U100) by 20% daily if the average blood glucose was greater than 140 mg/dL and decreasing by 20% in case of hypoglycemia (<70 mg/dL). The preprandial hyperglycemia correction dose was based on a table with three columns (insulin-sensitive, usual, and insulin-resistant), which could be adjusted to a more sensitive or resistant profile according to the researcher’s decision [1].
- Umpierrez et al., in their randomized clinical trial comparing basal-bolus insulin therapy using insulin analogs (detemir and aspart) versus human insulins (NPH and regular), proposed an insulin adjustment protocol increasing basal insulin doses (detemir and NPH) by 10% daily if fasting and pre-dinner blood glucose levels were between 140 and 180 mg/dL and by 20% if they were greater than 180 mg/dL. If fasting and pre-dinner blood glucose levels were between 70 and 99 mg/dL, basal insulin doses were reduced by 10%, and if any glucose level was <60 mg/dL, doses were reduced by 20%. The preprandial hyperglycemia correction dose was based on a table with three columns (insulin-sensitive, usual, and insulin-resistant), which could be adjusted according to the researcher’s decision [51].
- Umpierrez et al., in their randomized clinical trial comparing basal-bolus and basal-plus insulin therapy, conducted an insulin adjustment protocol, increasing the TDD of insulin by 10% daily if fasting and pre-dinner blood glucose levels were between 140 and 180 mg/dL and by 20% if they were greater than 180 mg/dL. If fasting and pre-dinner blood glucose levels were between 70 and 99 mg/dL, the TDD of insulin was reduced by 10%, and if any of these glucose levels were <70 mg/dL, the TDD of insulin was reduced by 20%. The preprandial hyperglycemia correction dose was based on a table with two columns, one for a more insulin-sensitive profile and the other for a usual profile, which could be adjusted according to the researcher’s decision [37].
- Pasquel et al., in a randomized clinical trial comparing the use of insulin analogs glargine U300 and glargine U100 in a hospital setting, adjusted doses daily to maintain fasting and pre-dinner blood glucose levels between 100 and 140 mg/dL. The TDD of insulin was increased by 10% if blood glucose levels were between 140 and 180 mg/dL, by 20% if between 180 and 240 mg/dL, and by 30% if greater than 240 mg/dL [47].
- Galindo et al., in a randomized clinical trial comparing the use of insulin analogs degludec and glargine U100 in a hospital setting, adjusted insulin doses daily to maintain fasting blood glucose below 140 mg/dL, pre-meal glucose levels below 180 mg/dL, and avoid hypoglycemia <70 mg/dL [48].
- Zhang et al., in a randomized clinical trial conducted in China, compared insulin dose adjustments using absolute TDD values with those according to TDD per patient weight (in IU/kg). Both algorithms were equally effective and safe in patients with type 2 diabetes in a hospital setting. In the weight-based dose adjustment group, the glargine U100 dose was titrated by 0.1 IU/kg/day if fasting blood glucose was greater than 140 mg/dL, and the total daily aspart dose was titrated by 0.1 IU/kg/day if postprandial glucose two hours after meals was greater than 180 mg/dL. For protocols using absolute dose adjustments, modified protocols by Riddle et al. [52] were used for basal insulin analog (glargine U100), and by Trence et al. [53] for prandial bolus insulin analog (aspart) [49].
- Li et al., part of the same group, had previously compared in a 2014 randomized controlled trial the adjustments of glargine U100 in absolute TDD values with those according to TDD per patient weight (in IU/kg), showing that both protocols were equally effective and safe [50].

### Patients with low acceptance of oral diet

**Table:** 

**R13:** IT SHOULD BE CONSIDERED omitting fixed bolus dose insulin doses in patients with poor acceptance of the oral diet	
IIa	C

#### Summary of evidence

- Magaji and Johnston (2011), in a narrative review and expert opinion article, recommend suspending bolus prandial insulin if the patient is not eating [46].
- Galindo et al. (2022), in a randomized clinical study comparing the use of degludec and glargine U100 insulins in a hospital setting, recommended pausing prandial insulin for patients with low food intake or fasting [48].

### Non-insulin agents in non-critical hospitalized patients

#### Insulin secretagogues

**Table:** 

**R14:** Insulin secretagogues (sulfonylureas and meglitinides) are NOT RECOMMENDED during hospital stays due to the risk of hypoglycemia. Insulin secretagogues may be reintroduced when the patient is clinically stable and discharge is imminent, provided no new contraindications have emerged	
III	**C**

#### Summary of evidence

- A case-control study by Rajendran et al. in the United Kingdom evaluated hospitalized patients using sulfonylureas and their risk of hypoglycemia. Hypoglycemic episodes occurred in 19% of patients, with higher risk in those over 65 years, on concurrent insulin therapy, or with a glomerular filtration rate (GFR) below 30 mL/min/1.73 m^2^. Sulfonylureas accounted for more than 30% of hypoglycemic episodes, more frequently than insulin-induced hypoglycemia, especially in the early morning hours [54].

### Biguanides (Metformin)

**Table:** 

**R15:** Metformin use to aid glycemic control in hospitalized patients on high doses of insulin MAY BE CONSIDERED when the acute condition is resolved, the patient is stable, GFR >30 mL/min/1.73 m^2^, and no contrast studies are planned	
IIb	**C**

#### Summary of evidence

- In a study by Bano et al., 387 out of 1800 patients continued metformin until the night before myocardial revascularization; there was no difference in lactic acidosis incidence between metformin users, non-users, and patients without diabetes [55].
- Salpeter et al., in a Cochrane meta-analysis of 357 clinical trials and cohort studies (70,490 patient-years for metformin, 55,451 patient-years for non-metformin), found the true incidence of lactic acidosis per 100,000 patient-years to be 4.3 cases for metformin users and 5.4 cases for non-users. However, this data does not pertain to hospitalized patients [56].
- Ma et al., in a systematic review and meta-analysis of observational studies on metformin use in COVID-19 patients, found reduced mortality risk for those previously on metformin but neutral effects for those who started it during hospitalization [57].

### Inhibitors of dipeptidyl peptidase 4 (DPP-4 inhibitors or gliptins)

**Table:** 

**R16:** DPP-4 inhibitors MAY BE CONSIDERED in non-critical patients with mild hyperglycemia (180–200 mg/dL), provided renal function is considered and the dose of the medication is adjusted accordingly	
IIb	A

#### Summary of evidence

- Vellanki et al. evaluated linagliptin use in a randomized clinical trial of 250 surgical patients, showing similar efficacy to basal-bolus therapy in patients with glucose levels below 200 mg/dL, with an 86% reduction in hypoglycemic events [58].
- Umpierrez et al., in a randomized study of 90 participants with well-controlled glucose, divided them into three groups: sitagliptin alone, sitagliptin plus basal insulin, and basal-bolus therapy. Hypoglycemia and hyperglycemia levels were similar, with reduced insulin use in the sitagliptin groups [59].
- Pasquel et al. studied 279 hospitalized type 2 diabetes patients using oral medications or insulin up to 0.6 IU/kg/day and glucose levels below 400 mg/dL. A multicenter randomized trial compared basal insulin plus sitagliptin to basal-bolus therapy. Outcomes showed no significant differences in daily glucose averages (171 ± 48.6 vs. 169.2 ± 48.6 mg/dL), hypoglycemia (9 vs. 12%), or treatment failure (16 vs. 19%) between the sitagliptin plus basal and basal-bolus groups, respectively [21].
- Gracia-Ramos et al., in a prospective, open-label randomized study of 76 hospitalized type 2 diabetes patients with admission glucose levels below 400 mg/dL and outpatient insulin doses below 0.5 IU/kg, divided patients into two groups: basal plus correction (BP) and basal plus correction with sitagliptin (s-BP). The s-BP group had lower average daily glucose (158.8 vs. 175.0 mg/dL, *p* = 0.014), a higher percentage of readings within the 70–180 mg/dL range (75.9 vs. 64.7%, *p* < 0.001), and fewer glucose readings >180 mg/dL (*p* < 0.001). The s-BP group also used fewer basal and supplemental insulin doses (*p* = 0.024 and *p* = 0.017, respectively) and had fewer daily insulin injections (*p* = 0.023). The rate of hypoglycemia was similar in both groups [60].
- Rabizadeh et al., in a meta-analysis of four randomized studies (n = 658), compared DPP-4 inhibitors (DPP4i) alone or combined with basal insulin versus basal-bolus therapy. There was no significant difference in average daily glucose levels between the groups (mean difference 4.63; 95% CI = −1.57, 10.83; *p* = 0.14) (I^2^ = 14%, *p* = 0.32). Total daily insulin dose was lower in the DPP4i group (mean difference −14.27; 95% CI = −22.47, −6.07; *p* = 0.001) (I^2^ = 92%, *p* = 0.001), as was the number of insulin injections (mean difference −0.79; 95% CI = −1.01, −0.57; p = 0.001) (I^2^ = 0%, *p* = 0.68). Hypoglycemia rates and treatment failure were not significantly different between the groups (RR 0.60, 95% CI = 0.34, 1.074; *p* = 0.08) (I^2^ = 37.3%, *p* = 0.18) and (RR 0.87, 95% CI = 0.64, 4.8; *p* = 0.38) (I^2^ = 49%, *p* = 0.11) respectively [61].
- Pérez-Belmonte et al. (2018) evaluated the real-world use of linagliptin in managing type 2 diabetes among inpatients in internal medicine departments. The study demonstrated that linagliptin is effective in achieving glycemic control with a low risk of hypoglycemia, particularly in patients with renal impairment, as it does not require dose adjustments based on renal function. These findings highlight linagliptin as a safe and practical option for hospitalized patients with type 2 diabetes, aligning with individualized treatment goals and inpatient care needs [22].
- Exception: Saxagliptin, linked to increased hospitalizations for heart failure in the SAVOR study, should be used with caution [36].
- DPP4i, such as sitagliptin and saxagliptin, require dose reductions in patients with moderate to severe renal impairment (e.g., creatinine clearance <50 mL/min), while linagliptin does not require dose adjustment due to its primarily non-renal excretion [62].

### Sodium-glucose cotransporter 2 (SGLT2) inhibitors

**Table:** 

**R17**: IT MAY BE CONSIDERED the continuation of SGLT2 inhibitors in non-critical hospitalized patients with type 2 diabetes, especially in those with heart failure. However, caution is advised around surgical procedures due to the potential risk of euglycemic diabetic ketoacidosis. SGLT2 inhibitors should generally be discontinued three days before surgery and resumed only when the patient is hemodynamically stable and normal eating has resumed	
IIb	B

**Table:** 

**R18:** In hospitalized patients using SGLT2 inhibitors, monitoring of ketones is RECOMMENDED, preferably by measuring capillary blood ketones or, if unavailable, urine ketones. Discontinue SGLT2 inhibitors if blood ketones are >1.5 mmol/L or if urine ketones are positive to reduce the risk of diabetic ketoacidosis	
I	C

#### Summary of evidence

- Singh et al. conducted a retrospective analysis of 5,936 hospitalizations in patients with type 2 diabetes who continued SGLT2 inhibitors during hospitalization and 30,569 who had them discontinued. Adjusting for severity, age, sex, BMI, ethnicity, insulin use, and surgical procedures, the continuation group had a 45% lower mortality risk (RR 0.55, 95% CI 0.42–0.73, *p* < 0.01), no increased risk of acute renal failure, and a slight reduction in hospital stay length (4.9 vs 4.7 days, RR 0.95, 95% CI 0.93–0.98, *p* < 0.01) [63].
- Bonora et al., in a review of 105 cases from the literature spanning May 2014 to April 2017, reported that euglycemic DKA (defined as glucose levels below 200 mg/dL) accounted for 35% of DKA cases in patients treated with SGLT2 inhibitors (SGLT2i) [64].
- Thiruvenkatarajan et al., in a systematic review, found that diet modification, especially reduced carbohydrate intake, was the primary risk factor for euglycemic and hyperglycemic DKA in patients using SGLT2i perioperatively. Hospitalized patients on SGLT2i should be clinically monitored for euglycemic DKA and have their ketone levels checked if they exhibit symptoms, even without significant hyperglycemia [65].
- Seki et al. conducted a systematic review analyzing 99 cases of perioperative ketoacidosis associated with SGLT2i. Their findings reinforced the critical need for a preoperative cessation period of at least three days to mitigate the risk of SGLT2i-associated perioperative ketoacidosis (SAPKA). Importantly, no cases of SAPKA were observed when SGLT2i were discontinued three or more days before surgery. The study also highlighted key risk factors for SAPKA, including perioperative fasting, surgical stress, and inadequate perioperative fluid management. The authors noted that bariatric and coronary artery bypass surgeries were the most commonly associated procedures, emphasizing the need for vigilance in high-risk surgical settings. They concluded that while the 2020 U.S. Food and Drug Administration (FDA) guideline recommending a preoperative cessation of 3–4 days appears effective, further research is required to identify additional risk factors and optimize perioperative management strategies for patients on SGLT2i [66].
- Umapathysivam et al., in a retrospective cohort study, investigated the resolution time of DKA in patients with type 2 diabetes treated with SGLT2i. Their study found that the resolution of SGLT2i-associated DKA was significantly delayed compared to type 1 diabetes (T1D) DKA. Specifically, the median time to resolution of ketosis and acidosis in patients with SGLT2i-DKA was 36 h (IQR: 24–72), compared to 18 h (IQR: 12–27) in T1D-DKA (*p* = 0.002). This delay was associated with lower insulin administration in the first 24 h among the SGLT2i-DKA group (median: 44 units) compared to the T1D-DKA group (median: 87 units, *p* = 0.01). The authors suggest that the pathophysiology of SGLT2i-DKA, characterized by relatively normal or mildly elevated plasma glucose levels, may contribute to differences in treatment responses. This tendency toward euglycemia could result in inadequate insulin dosing when using standard DKA protocols, leading to prolonged ketosis and delayed recovery. Adjustments to insulin and dextrose infusion protocols may be necessary to optimize the management of SGLT2i-DKA [67].
- Sebastian-Valles et al., in a retrospective cohort study, demonstrated that DKA in patients with type 1 and type 2 diabetes treated with SGLT2i tends to exhibit a more rapid and aggressive onset compared to matched controls not receiving SGLT2i. Their study also highlighted a higher risk of admission to ICU among these patients, underscoring the severe clinical course often associated with SGLT2i-DKA [68].
- Kosiborod et al., in the multicenter randomized DARE-19 trial, evaluated dapagliflozin against placebo in 1,250 hyperglycemic hospitalized COVID-19 patients. There was no significant difference in organ dysfunction or death between the groups. DKA occurred in 0.32% of the treatment group and 0% of the placebo group, without statistical significance [69].
- Voors et al., in the EMPULSE trial, studied empagliflozin in 530 hospitalized heart failure patients regardless of hyperglycemia. The primary outcome of the study was clinical benefit (death from any cause, number of heart failure events, time to the first heart failure event, or a 5-point or greater change from baseline in symptom score). More patients treated with empagliflozin experienced clinical benefits compared to placebo, although empagliflozin was only started after clinical stabilization, not in the acute phase [70].
- Biegus et al., also in the EMPULSE trial, assessed empagliflozin in heart failure patients post-stabilization, regardless of hyperglycemia. Compared to placebo, those treated with empagliflozin showed greater reductions in decongestion markers at all time points. Adjusted mean differences in weight loss at days 15, 30, and 90 were significant, and higher hematocrit on day 15 was linked to a significantly greater probability of clinical benefit at day 90 (hierarchical composite of all-cause mortality, heart failure events, and a 5-point or greater change from baseline in the total symptom score of the Kansas City Cardiomyopathy Questionnaire (change from baseline to 90 days)) [71].
- Okoroike et al., in the INSIGHT-HF study, a retrospective analysis of 2,663 hospitalized patients over 18 years old with left ventricular ejection fraction ≤40%, showed that the 30-day prescription rates were significantly higher for those who started SGLT2i at discharge compared to those who did not initiate them during hospitalization (96 vs 14.7%, *p* < 0.0001). The 30-day heart failure readmission rate was significantly lower for those who started SGLT2i before discharge (9.3 vs 22.7%, *p* = 0.04), though cardiovascular mortality was not significantly different between groups (4 vs 10.7%, *p* = 0.21), highlighting the importance of medication reconciliation and the initiation of outpatient treatment at discharge [72].
- Chieng et al., in a meta-analysis of 1,758 patients who received SGLT2i within two weeks of hospital admission, found no significant clinical complications. In a sub-analysis of patients hospitalized for heart failure decompensation, SGLT2i were associated with a 27% relative risk reduction for readmission due to heart failure compared to placebo, although use was not during the acute phase [73].
- Huang et al., in a retrospective study, reported that SGLT2i did not increase the likelihood of significant ketonemia compared to DPP-4 inhibitors, suggesting safety when appropriately indicated in a hospital setting [74].

### Glucagon-like peptide-1 (GLP-1) agonists

**Table:** 

**R19:** GLP-1 agonists are NOT RECOMMENDED for use in non-critical hospitalized patients who are undergoing procedures involving sedation or anesthesia due to the potential for interference with gastric emptying	
III	C

#### Summary of evidence

- While GLP-1 agonists appear to improve glycemic control in hospitalized patients with lower insulin doses needed compared to conventional insulin regimens [75], their routine use is not recommended. This is particularly true for patients undergoing procedures requiring general anesthesia or sedation due to the potential risk of aspiration. Several studies, especially involving dulaglutide and semaglutide, have linked GLP-1 agonists to higher risks of gastric residual accumulation during upper endoscopy or gastric ultrasound [76–79].
- A recent case–control study with 446 patients assessed colon preparation efficacy in colonoscopies. Patients using GLP-1 agonists had worse colon preparation (15.5 vs. 6.6%, *p* = 0.01) and a higher need for repeat colonoscopies due to inadequate preparation (18.9 vs. 11.1%, *p* = 0.041) [80].
- Given the lack of randomized studies in this population and the potential risks, GLP-1 receptor agonists should be reserved for discharge planning and special cases.

### Technologies and glucose sensor in non-critical hospitalized patients

**Table:** 

**R20**: The use of continuous glucose monitoring (CGM) in a hospital setting MAY BE CONSIDERED for non-critical inpatients. Limitations must be taken into account	
IIb	B

#### Summary of evidence

- Spanakis et al., in a randomized clinical trial with 185 patients, demonstrated that CGM use in medical and surgical patients (including vascular, orthopedic, general, thoracic, and other surgeries) with type 1 or type 2 diabetes is safe and effective for guiding insulin therapy. It is not inferior to capillary blood glucose monitoring in terms of time in the target range of 70–180 mg/dL and average daily glucose, significantly reducing recurrent hypoglycemic events: recurrence of hypoglycemia (1.80 ± 1.54 vs. 2.94 ± 2.76 events/patient; *p* = 0.03), lower percentage of time below <70 mg/dL (1.89% ± 3.27 vs. 5.47% ± 8.49; *p* = 0.02), and lower incidence rate ratio <70 mg/dL (0.53 [95% CI 0.31–0.92]) and <54 mg/dL (0.37 [95% CI 0.17–0.83]) [100].
- Other studies have highlighted technological limitations that reduce the accuracy of subcutaneous sensors, such as the delay between interstitial fluid and blood glucose measurements and interference from substances like acetaminophen, dopamine, heparin, mannitol, maltose, ascorbic acid, uric acid, and salicylic acid [81, 82].
- In a 2023 narrative review, Clubbs-Coldron et al. reviewed existing literature and noted that while some studies show benefits in certain metrics, such as improved glycemic control, reduced hospital stay, and decreased risk of severe hypoglycemia or hyperglycemia, there is a lack of data on clinical and financial outcomes and the impact on healthcare team workflows. However, patients with diabetes can be empowered to better self-manage their condition with direct access to their glucose data [83].
- Hagerf et al., in a prospective study comparing 1,546 CGM Dexcom G6 readings with capillary and plasma glucose readings in 61 patients post-solid organ transplant or abdominal surgeries (total or partial pancreatectomies), found good sensor accuracy with a mean absolute relative difference (MARD) of 9.4% compared to plasma glucose, and 98.9% of glucose readings within zones A and B of the Error Grid [84].

**Table:** 

IMPORTANT NOTE 11: Use of Continuous Glucose Monitors (CGM) in Non-Critical Hospitalized Patients
• The use of CGM may require additional capillary blood glucose tests when blood glucose levels are <85 mg/dL or >300 mg/dL, if hypoglycemia symptoms are present, in cases of hemodynamic instability, device reading failures, or during the immediate postoperative period
• For patients already using CGMs, maintaining their use during hospitalization can improve patient satisfaction [85, 86]
• In 2022, Matievich et al. demonstrated the functional integrity of the Freestyle Libre CGM in simulations of X-rays, computerized tomography scans, and magnetic resonance imaging (MRI), confirming their safety during these procedures. However, it is important to note that these sensors do not have an external transmitter unit, which could pose a problem during MRI scans [87]. The Freestyle Libre package insert recommends removing the sensor for MRI exams
• Severe anemia (Hb <7 g/dL) can affect CGM accuracy. It is also important to understand each device to identify other potential interferences (ascorbic acid, acetaminophen, acetylsalicylic acid) and whether calibration is required. In case of doubt, consult the product’s technical support. Another critical point is that CGM sensors generally lose accuracy in hypoglycemic ranges, requiring confirmation with capillary blood glucose tests [88]
• Hospitals should have institutional protocols for CGM management as part of the training for glycemic control.
• CGM sensors can be used during hospitalization, exams, and procedures, but extreme values must be confirmed by capillary blood glucose tests, especially in the first 24–48 h post-surgery
• In unstable or critically ill patients, the likelihood of discrepancies and loss of accuracy increases, necessitating more frequent capillary blood glucose tests and potential replacement of CGM readings
• Given the healthcare reality in Brazil, where access to and the cost of sensors remain high, CGM use is typically limited to patients who are already using it upon admission or wish to start using it during their hospital stay. For these cases, there are no contraindications, and it is not necessary to remove the sensors during hospitalization, even for surgical procedures, as long as the sensor is not placed in the surgical site

**Table:** 

IMPORTANT NOTE 12: Management of Continuous Subcutaneous Insulin Infusion (CSII) Systems in Hospitalized Patients
• Continuous Subcutaneous Insulin Infusion (CSII) systems are devices that deliver insulin continuously under the skin. They can be fully programmed manually or have varying degrees of automation (AID). Automated systems can also be set to manual mode if needed [89]
• As the use of CSII becomes more popular, healthcare providers will need to manage hospitalized patients on CSII therapy. When patients are admitted, a decision must be made on whether they can continue using CSII. This decision depends on the patient’s ability to safely operate the CSII and the familiarity of healthcare providers with the system [90]
• If there are no knowledgeable individuals available to manage the CSII, it is recommended to switch to another effective insulin regimen until the patient or their family can resume its use [89]
• Contraindications for CSII Use in Hospitalized Patients:
○ Impaired level of consciousness
○ Inability of the patient to correctly demonstrate basic pump settings
○ Critical illness requiring intensive care
○ Psychiatric conditions affecting the patient’s ability to manage the system
○ Patients at risk of suicide
○ Diabetic ketoacidosis and hyperglycemic hyperosmolar nonketotic syndrome
○ Patient’s refusal or unwillingness to participate in self-care
○ Lack of pump supplies
○ Lack of trained healthcare professionals, diabetes educators, or specialists
○ Decision by the healthcare team
• The effective insulin regimen for such patients is the basal-bolus regimen with multiple daily injections
• Calculate the total average insulin dose used with the CSII over the most recent period (e.g., the last seven days); this information can be obtained from the patient or directly from the pump
○ Prescribe 50% of the total average insulin dose as basal insulin, which can be NPH insulin or a long-acting analogue (e.g., glargine)
○ For bolus insulin at meals, prescribe 50% of the total average insulin dose, divided into three or more pre-meal doses of regular insulin or a rapid-acting analogue (e.g., lispro or aspart)
○ Adjust the doses according to the above recommendations
• A safety adjustment (e.g., a 30% reduction) can be made to minimize the risk of hypoglycemia in high-risk situations, such as in patients already experiencing hypoglycemia or with restricted oral intake. However, generally, lower doses of insulin are used with CSII compared to basal-bolus therapy, so this reduction is often unnecessary
• Turn off the CSII approximately two hours after the basal insulin injection
• **Practical Example:**
If a patient’s total average insulin dose used with CSII over the past seven days is 48 units/day:
○ Basal Insulin: 50% of 48 units = 24 units. Prescribe 24 units/day of insulin glargine or 8 units three times a day of NPH insulin
○ Bolus Insulin: 50% of 48 units divided by three meals = 8 units of rapid-acting insulin at each meal
• The CSII can be restarted when the patient or a family member is capable of managing it or upon hospital discharge

### Correction and monitoring of hypoglycemia

**Table:** 

**R21:** It is RECOMMENDED to correct hospital-acquired hypoglycemia levels 1 (54–70 mg/dL) and 2 (<54 mg/dL) orally, enterally, or via gastrostomy with a solution containing 15–30 g of glucose (0.3 g/kg). Blood glucose should be reassessed every 15 min, repeating the treatment until blood glucose levels are above 100 mg/dL	
I	**B**

**Table:** 

**R22:** It is RECOMMENDED to correct hypoglycemia intravenously in cases of level 3 hypoglycemia (blood glucose <70 mg/dL associated with altered consciousness) and/or when oral, enteral, or gastrostomy routes are not feasible	
I	**C**

**Table:** 

**R23:** In the event of level 3 hypoglycemia (blood glucose <70 mg/dL with altered consciousness) without venous access, it is RECOMMENDED to administer 1 mg of glucagon intramuscularly or subcutaneously	
I	**C**

#### Summary of evidence

- Hypoglycemia treatment requires the ingestion of glucose or carbohydrate-containing foods, with glucose being the preferred choice. The acute glycemic response correlates better with glucose intake than with the carbohydrate content of the food. Adding fats can delay and prolong the glycemic response, while protein intake in patients with type 2 diabetes may increase insulin response without raising blood glucose levels [91].
- McTavish et al. demonstrated in a randomized controlled trial that correcting hypoglycemia is more effective with oral administration of 0.3 g of carbohydrate per kg of body weight in children and adults with type 1 diabetes on continuous insulin infusion pumps [92].
- Georgakopoulos et al. showed in a comparative study with healthy volunteers experiencing subcutaneous insulin-induced hypoglycemia that glucose supplementation was more effective than sucrose in reversing hypoglycemia [93].
- McTavish and Wiltshire found that carbohydrate supplementation (0.3 g/kg) effectively corrected blood glucose levels within 15 min in children with type 1 diabetes during camp [94].
- Namba et al. reported that in a pharmacokinetic and pharmacodynamic study of intramuscular or intravenous glucagon, blood glucose levels increased by an average of 55 mg/dL within 20 min after intramuscular administration, offering an alternative when intravenous glucose is not available [95].

**Table:** 

**IMPORTANT NOTE 13: Use of Intravenous Glucose Solutions**
• Intravenous correction of hypoglycemia should be reserved for severe cases and/or when oral/enteral routes are not feasible, to avoid overcorrection of blood glucose and thrombophlebitis. If intravenous correction is necessary via a peripheral vein, solutions of 25–50% glucose should be used, with a total of 10–30 g of glucose ((100—capillary glucose) * 0.2)—or 10 g for level 1 hypoglycemia and 20–30 g for levels 2 or 3), administered as a slow bolus. Blood glucose should be reassessed every 15 min until levels exceed 100 mg/dL. Each 10 mL of 50% glucose solution contains 5 g of glucose, and each 10 mL of 25% glucose solution contains 2.5 g

### Summary table of recommendations

**Table:** 

RECOMMENDATIONS	Class	Level
**Definition of Hospital Hyperglycemia:** R1: It is RECOMMENDED to use the criterion of HOSPITAL HYPERGLYCEMIA for all individuals with capillary or plasma glucose levels above 140 mg/dL, regardless of the prior existence of diabetes, as it correlated with worse outcomes	I	B
**Screening:** R2: It is RECOMMENDED to screen for hospital hyperglycemia with a capillary or plasma glucose test in all adult inpatients upon admission, regardless of prior diabetes diagnosis	I	C
**HbA1c Testing:** R3: It is RECOMMENDED to measure HbA1c in all patients with confirmed hospital hyperglycemia or pre-existing diabetes, provided testing has not been done in the past three months. This assists in diagnosing previously undetected diabetes when HbA1c is above 6.5%, and supports discharge planning	I	B
**Glycemic Monitoring:** R4: It is RECOMMENDED to monitor capillary glucose levels in all patients with hospital hyperglycemia, diabetes, or risk factors	I	C
**Suspected Diabetic ketoacidosis:** R5: It is RECOMMENDED to test for capillary ketonemia or ketonuria in the following situations: blood glucose levels above 200 mg/dL and signs and symptoms suggestive of ketoacidosis; or patients on SGLT2 inhibitors with signs and symptoms of ketoacidosis, even with normal blood glucose levels (euglycemic diabetic ketoacidosis)	I	C
**Glucocorticoid-Induced Hyperglycemia:** R6: In patients using glucocorticoids, regardless of a prior diagnosis of diabetes mellitus, IT IS RECOMMENDED to perform capillary glucose measurements upon waking and before dinner	I	C
**Glycemic Targets:** R7: In non-critical patients with or without diabetes, it is RECOMMENDED to aim for glycemic control targets between 100–180 mg/dL to avoid hyper- and hypoglycemia	I	B
**Basal-plus and Basal-bolus Insulin Regimen:** R8: Scheduled basal insulin combined with pre-prandial bolus IS RECOMMENDED for treating persistent hyperglycemia in non-critical hospitalized patients. This approach is associated with better glycemic control, reduction of adverse outcomes, and shorter hospital stays	I	A
**Insulin Therapy for Inpatients for Glucocorticoid-Induced Hyperglycemia:** R9: IT MAY BE CONSIDERED to use a single daily dose of NPH insulin in the morning for the treatment of hyperglycemia secondary to glucocorticoid use in patients with predominantly afternoon hyperglycemia, starting at 0.1 IU/kg/day, either alone or in combination with other forms of insulinization or oral medications	IIb	B
**Insulin Therapy Adjustments #1:** R10: It is RECOMMENDED to adjust insulin therapy every 24–48 h, according to blood glucose monitoring, dietary status, and the dosage of medications with hyperglycemic effects	I	C
**Insulin Therapy Adjustments #2:** R11: It is RECOMMENDED that in a basal-bolus regimen, the adjustment of the total daily insulin dose be based on the average blood glucose level or fasting and pre-meal blood glucose values. In cases where individualization of basal or prandial doses per meal is required, it is suggested to consult a team specialized in glycemic control (e.g., endocrinologists or diabetologists)	I	B
**Insulin Therapy Adjustments #3:** R12: It is RECOMMENDED to adopt a standardized approach for adjusting insulin doses, which can be equally effective using percentage values of the total daily dose (TDD) of insulin (10–20% per day) or according to the TDD of insulin per patient weight (0.05–0.1 IU/kg/day)	I	B
**Insulin Therapy in Patients with Low Acceptance of Oral Diet:** R13: It SHOULD BE CONSIDERED omitting fixed bolus dose insulin doses in patients with poor acceptance of the oral diet	IIa	C
**Insulin Secretagogues:** R14: Insulin secretagogues (sulfonylureas and meglitinides) are NOT RECOMMENDED during hospital stays due to the risk of hypoglycemia. Insulin secretagogues may be reintroduced when the patient is clinically stable and discharge is imminent, provided no new contraindications have emerged	III	C
**Metformin:** R15: Metformin use to aid glycemic control in hospitalized patients on high doses of insulin MAY BE CONSIDERED when the acute condition is resolved, the patient is stable, GFR >30 mL/min/1.73 m^2^, and no contrast studies are planned	IIb	C
**DPP-4 Inhibitors:** R16: DPP-4 inhibitors MAY BE CONSIDERED in non-critical patients with mild hyperglycemia (180–200 mg/dL), provided renal function is considered and the dose of the medication is adjusted accordingly	IIb	A
**SGLT2 Inhibitors #1:** R17: It MAY BE CONSIDERED the continuation of SGLT2 inhibitors in non-critical hospitalized patients with type 2 diabetes, especially in those with heart failure. However, caution is advised around surgical procedures due to the potential risk of euglycemic diabetic ketoacidosis. SGLT2 inhibitors should generally be discontinued three days before surgery and resumed only when the patient is hemodynamically stable and normal eating has resumed	IIb	B
**SGLT2 Inhibitors #2:** R18: In hospitalized patients using SGLT2 inhibitors, monitoring of ketones is RECOMMENDED, preferably by measuring capillary blood ketones or, if unavailable, urine ketones. Discontinue SGLT2 inhibitors if blood ketones are >1.5 mmol/L or if urine ketones are positive to reduce the risk of diabetic ketoacidosis	I	C
**GLP-1 Agonists:** R19: GLP-1 agonists are NOT RECOMMENDED for use in non-critical hospitalized patients who are undergoing procedures involving sedation or anesthesia due to the potential for interference with gastric emptying	III	C
**CGM:** R20: The use of continuous glucose monitoring (CGM) in a hospital setting MAY BE CONSIDERED for non-critical inpatients. Limitations must be taken into account	IIb	B
**Hypoglycemia #1:** R21: It is RECOMMENDED to correct hospital-acquired hypoglycemia levels 1 (54–70 mg/dL) and 2 (<54 mg/dL) orally, enterally, or via gastrostomy with a solution containing 15–30 g of glucose (0.3 g/kg). Blood glucose should be reassessed every 15 min, repeating the treatment until blood glucose levels are above 100 mg/dL	I	B
**Hypoglycemia #2:** R22: It is RECOMMENDED to correct hypoglycemia intravenously in cases of level 3 hypoglycemia (blood glucose <70 mg/dL associated with altered consciousness) and/or when oral, enteral, or gastrostomy routes are not feasible	I	C
**Hypoglycemia #3:** R23: In the event of level 3 hypoglycemia (blood glucose <70 mg/dL with altered consciousness) without venous access, it is RECOMMENDED to administer 1 mg of glucagon intramuscularly or subcutaneously	I	C

## Data Availability

No datasets were generated or analysed during the current study.
